# Converting Health Level 7 Clinical Document Architecture (CDA) documents to Observational Medical Outcomes Partnership Common Data Model (OMOP CDM) by leveraging CDA Template definitions

**DOI:** 10.1093/jamiaopen/ooaf022

**Published:** 2025-03-26

**Authors:** Florian Katsch, Rada Hussein, Tanja Stamm, Georg Duftschmid

**Affiliations:** Center for Medical Data Science, Medical University of Vienna, 1090 Vienna, Austria; Ludwig Boltzmann Institute for Digital Health and Prevention, 5020 Salzburg, Austria; Ludwig Boltzmann Institute for Digital Health and Prevention, 5020 Salzburg, Austria; Center for Medical Data Science, Medical University of Vienna, 1090 Vienna, Austria; Center for Medical Data Science, Medical University of Vienna, 1090 Vienna, Austria

**Keywords:** OMOP, HL7 CDA, HL7 Templates, Health Information Interoperability

## Abstract

**Objectives:**

This work aims to develop a methodology for transforming Health Level 7 (HL7) Clinical Document Architecture (CDA) documents into the Observational Medical Outcomes Partnership (OMOP) Common Data Model (CDM). The described method seeks to improve the Extract, Transform, Load (ETL) design process by using HL7 CDA Template definitions and the CDA Refined Message Information Model (CDA R-MIM).

**Material and Methods:**

Our approach utilizes HL7 CDA Templates to define structural and semantic mappings. Supported by the CDA R-MIM for semantic alignment with the OMOP CDM, we developed a tool named CDA Rabbit that enables the generation of Rabbit-In-a-Hat project files from HL7 CDA Template definitions and could be successfully integrated into the existing toolchain around OMOP.

**Results:**

We tested our approach using 13 CDA Templates from the Austrian national EHR System (ELGA) and 430 anonymized CDA test documents that were mapped to 10 OMOP CDM tables. The data quality assessment, using OMOP’s DataQualityDashboard, showed a 99% pass rate, indicating a robust and accurate data transformation.

**Conclusion:**

This study presents a novel framework for transforming HL7 CDA documents into OMOP CDM using template definitions and CDA R-MIM. The methodology improves semantic interoperability, mapping reusability, and ETL design efficiency. Future work should focus on automating code generation, improving data profiling, and addressing cyclic dependencies within CDA templates. The presented approach supports improved secondary use of health data and research while adhering to standardized data models and semantics.

**Discussion:**

Using CDA Templates for ETL design addresses common ETL challenges, such as data accessibility during ETL design, by decoupling the process from the actual CDA instances. Future work could focus on extending this approach to automatically generate boilerplate code, address cyclic dependencies within CDA Templates, and adapt the method for the use with FHIR profiles.

## Introduction

In health care, the digitization of clinical information has ushered in a new era of data-driven decision making, improved secondary research data availability and ultimately better patient outcomes.^1^ Considerable progress has been made toward these goals with numerous initiatives in the areas of interorganizational, transnational data exchange and the European Health Data Space for secondary use,^2^ which is taking shape as a truly pan-European project to enable cross-border data access for research. In addition to organizational and legal barriers, there are still technical challenges to overcome, especially in the area of semantic interoperability. A common challenge is the transformation of routine data into research-oriented common data models while preserving the original semantics.

The Clinical Document Architecture (CDA),^3^ developed by Health Level 7 (HL7), has been adopted in a number of countries as the standard for structuring and exchanging clinical documents in electronic form. Clinical Document Architecture provides a standardized framework and format for representing health-related information, and is the most widely used content standard for routine data.^4^^,^^5^

In the area of secondary use of health data, the Observational Medical Outcomes Partnerships (OMOP) Common Data Model (CDM) has gained prominence.^6^^,^^7^ The OMOP CDM is designed to harmonize and standardize health data by imposing a common structure and terminologies, making it suitable for large-scale analysis and research across organizational boundaries.^8^ Further, it has proven to be useful in, for example, cohort identification,^9^ clinical trial recruitment^10^ and federated machine learning applications.^11^ Observational Health Data Sciences and Informatics (OHDSI) provides a rich software toolset to utilize the OMOP CDM.

Although the HL7 CDA itself can be interpreted as a common data model, it is not the ideal format for direct use in scientific analysis. Firstly, the document-based representation makes it difficult to process fine-grained information items, such as drug administrations or diagnoses, because they must first be decomposed from their document container.^12^ Secondly, there is a lack of open source tools for secondary analysis of CDAs. Both limitations do not exist for the OMOP CDM. In order to make CDA-based routine data accessible for large-scale clinical research, it thus seems essential to transform them into the OMOP CDM.

However, transforming CDA-formatted clinical documents into OMOP CDM is a multifaceted challenge. The inherent complexity and variability in the structure of CDA documents requires a thorough Extract-Transform-Load (ETL) design process.^12^^,^^13^ Unfortunately, the current OHDSI ETL toolset only considers relational databases as data source and the CDA format is not supported.^14^

The feasibility of transforming CDA to OMOP CDM has been demonstrated previously.^12^^,^^15–19^ However, to the best of our knowledge, OHDSI’s toolset cannot be fully utilized for CDA sources. The identified gap is often circumvented by transforming CDA documents to an intermediate relational database.^12^^,^^17^^,^^18^ Our approach attempts to close this gap by enabling ETL design directly upon CDA and related standards.

Pecoraro et al demonstrated how structured CDA documents and references to the CDA Refined Message Information Model (R-MIM) can be utilized to define ETL processes.^20^ We extend this idea by considering also CDA Templates and apply it to the OMOP CDM as the target of the transformation. The HL7 Templates Standard^21^ provides a language for defining the clinical content, semantics, and structure of HL7 documents. The present paper explores a methodology that leverages the rich semantics contained within CDA Templates and the CDA R-MIM to facilitate the transformation of CDA formatted clinical documents into OMOP CDM. By exploiting the inherent relationships within CDA Templates and aligning them with the OMOP CDM framework, we aim to provide additional guidance and streamline the ETL design process. This approach further supports the characterization of source data and provides guidance for the vocabulary mapping stage, which together account for over half of the time spent in ETL development processes.^22^

We present a method that allows CDA structures and terminology to be mapped to the OMOP CDM by extracting relevant information directly from CDA Templates. Furthermore, we provide an alternative to the WhiteRabbit tool that prepares CDA Templates for use with subsequent tools within the OHDSI toolchain. This approach benefits from the existing OHDSI tool ecosystem and adopts the common approach to ETL design imposed by the OHDSI community. Our tool is publicly available from our GitLab repository: https://mimapps.meduniwien.ac.at/gitlab/omop/cdarabbit.

### OMOP CDM and OHDSI tools

**Figure 1.**
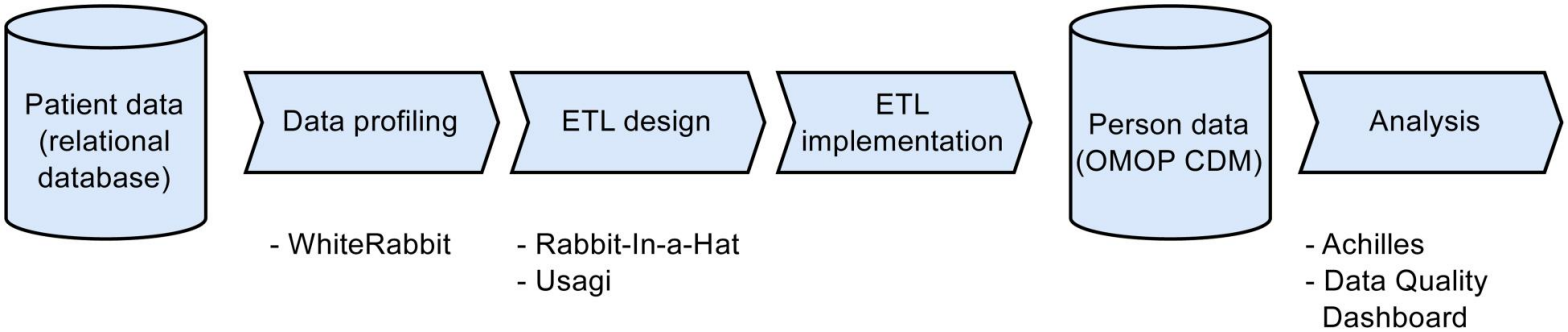
The overall OMOP ETL implementation process and OHDSI’s open source tools used in these steps. Data profiling of the source relational databases is supported by WhiteRabbit and results in a scan report that is used by Rabbit-In-a-Hat to perform the structural ETL design. The vocabulary mapping process is supported by OHDSI’s Usagi tool. Several tools exist for data quality management and analysis of the OMOP CDM data, which may be applied for further refinement of the ETL design. Abbreviations: CDM, Common Data Model; OHDSI, Observational Health Data Sciences and Informatics; OMOP, Observational Medical Outcomes Partnership.

The OMOP CDM forms the core of the OMOP approach of supporting observational health research on diverse health data sources. The data model, in its current version (v5.4^23^) consists of 39 tables capturing, for example, demographics, medication use, and health conditions. In addition to tables for routine clinical data, some tables are reserved for metadata, vocabularies, and relationships between concepts. For each table and its fields, a common understanding of their semantic content, used format and possible relationships has been established by the OHDSI community. This includes the definition of “standard” vocabularies for certain facts, for example, laboratory measurements are coded as Logical Observation Identifiers Names and Codes (LOINC), whereas diagnoses are captured as Systematized Nomenclature of Medicine Clinical Terms. In order to achieve consistent data and thus allow analyses across different data sources, the latter have to transform their local data models and terminologies to the OMOP CDM. An overview of this process is depicted in Figure 1. To support this transformation, OHDSI provides an ecosystem of open-source tools:

- WhiteRabbit^14^ is used as a data profiling tool, scanning the source relational database, and characterizing its structure and content.
- Using output from WhiteRabbit, Rabbit-In-a-Hat assists in the process of structurally mapping source data elements to the CDM by graphically connecting source data structures to CDM tables and fields. In addition, comments and logic statements can be added in free text. The resulting ETL definition document is then refined and guides the subsequent ETL implementation process. It often represents a milestone deliverable in ETL projects.
- While Rabbit-In-a-Hat is used to define structural mappings, Usagi is used for vocabulary mappings. The use of standard vocabularies requires the mapping of source concepts to concepts within these standard vocabularies. Usagi supports this process by suggesting mappings based on term similarity.
- Once the ETL process has been implemented and the data have been transformed, tools for assessing the quality of this process can be applied to further refine the ETL process and to check the compliance with certain quality criteria (Achilles, DataQualityDashboard).

### HL7 CDA, Templates, and CDA R-MIM

HL7 Version 3 CDA is a widely adopted standard for the exchange of routine clinical documents in electronic health records (EHRs) and other health care-related systems. Clinical Document Architecture documents are designed to capture structured and unstructured data in the form of persistent documents. They represent patient-centered information, are designed to be human-readable, and support semantic interoperability by structuring and annotating information elements at different levels of granularity. Clinical Document Architecture documents are encoded in Extensible Markup Language (XML) format.

The CDA R-MIM is a specific instantiation of the Reference Information Model (RIM). The latter represents the foundational model for all HL7 Version 3 standards by defining the conceptual framework through abstract core classes. Essentially, the CDA R-MIM specifies a generic data model for any type of clinical document.

**Figure 2.**
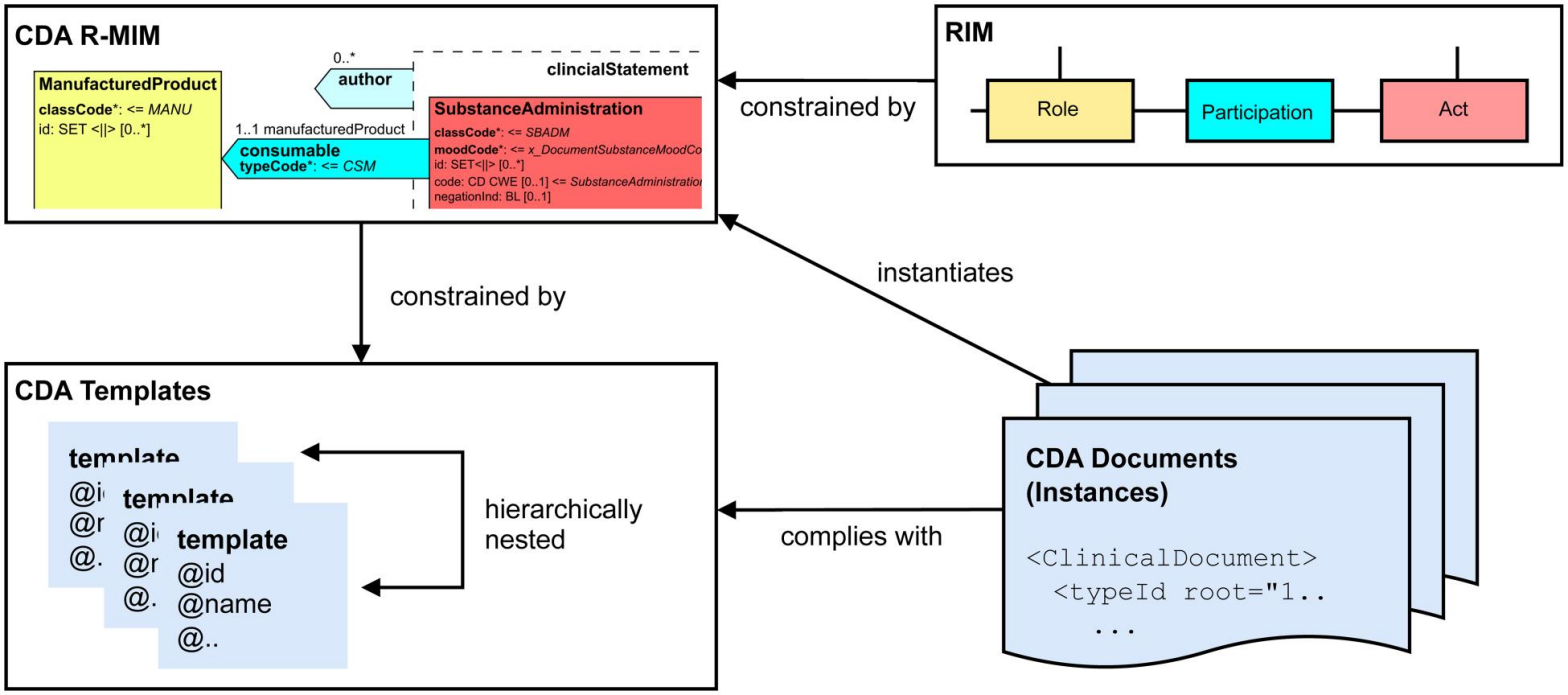
Clinical Document Architecture (CDA) documents instantiate the CDA R-MIM according to the structural prescriptions of the underlying document level CDA Templates, which reference CDA Templates at more granular levels. The CDA templates in turn constrain the underlying CDA R-MIM model, which is itself a constrained derivation of the RIM meta-model. Abbreviations: R-MIM, Refined Message Information Model.

Clinical Document Architecture Templates allow to specify a specific data model for a particular type of clinical document by constraining the CDA R-MIM. As an example, they define which components of the CDA R-MIM are required, optional or to be omitted for a laboratory report. This multimodel approach allows the definition of document types for specific domains and use cases. Ensuring conformance of CDA documents to a particular template definition supports semantic interoperability within a defined scope. This conformance can be checked automatically by validating a CDA document against the corresponding template definitions (see Figure 2).

The HL7 Template Standard^21^ defines a template as (1) a set of metadata to identify, version and capture the purpose of a template and (2) the body, consisting of a set of constraints on a model. This work focuses on templates that constrain the CDA R-MIM model to describe CDA documents, hence we refer to these templates as CDA Templates. Four types of CDA templates are particularly relevant to this work. Document level CDA Templates constrain CDA header fields and define the overall structure of a CDA document via nested section level Templates. Section level CDA Templates constrain elements and attributes within a section and may contain entry level CDA Templates. The latter are used, for example, to define a representation of clinical observations. The nesting of CDA Templates allows a hierarchical template structure to be built and existing template definitions to be reused in different contexts. Clinical Document Architecture Templates are used to define a comprehensive repository of reusable CDA components for a given domain. They can also be used to generate XML Schema and Schematron definitions, against which CDA document instances can be validated and represent the computable basis of implementation guides.

## Materials and methods

The CDA Templates used in this work were created in the context of the Austrian national EHR System (ELGA)^24–26^ and are publicly available from the ELGA CDA Template repository.^27^ The contained ELGA template definitions are represented in XML format and follow the HL7 Template Standard. Our methodology is applicable to any CDA data source that employs the HL7 Template Standard.

In order to map CDA documents to the OMOP CDM, we first extract information from the underlying CDA Template definitions. This is followed by traversing hierarchically nested CDA Templates and identifying the CDA R-MIM classes referenced by the templates. Finally, we prepare our output to be processable by OHDSI’s Rabbit-In-a-Hat tool.

The hierarchical template structure is decomposed by originating from the document level CDA Templates and then descending into their template definition “tree.” This traversal identifies all possible mapping sources and gathers structural and semantic information about the template content and relationships to other CDA templates. For each template found, the following information is extracted:

- Metadata: identifiers, status, level, referenced CDA R-MIM class, description.
- Locators: all possible locations where this template might appear within a CDA instance, expressed as XML Path Language (XPath) expressions.
- Elements: CDA elements defined by a template are extracted with relative location, description, and properties (eg, cardinality, type, vocabulary binding).

Template definitions explicitly refer to the CDA R-MIM class, which they constrain. Based on the semantics of the CDA R-MIM class, likely mapping targets in the OMOP CDM can be derived and suggested. These suggestions may be further refined in the mapping process. As a trivial example, CDA Templates referring to the *Patient* class will typically be mapped to the CDM *person* table, and *Patient’*s element *administrativeGenderCode* will typically be mapped to the *person’*s attribute *gender_source_value*. These “default” mappings are only derived from the semantics of the CDA R-MIM classes and thus serve as a useful first mapping suggestion regardless of the domain in which a template is used.

**Figure 3.**
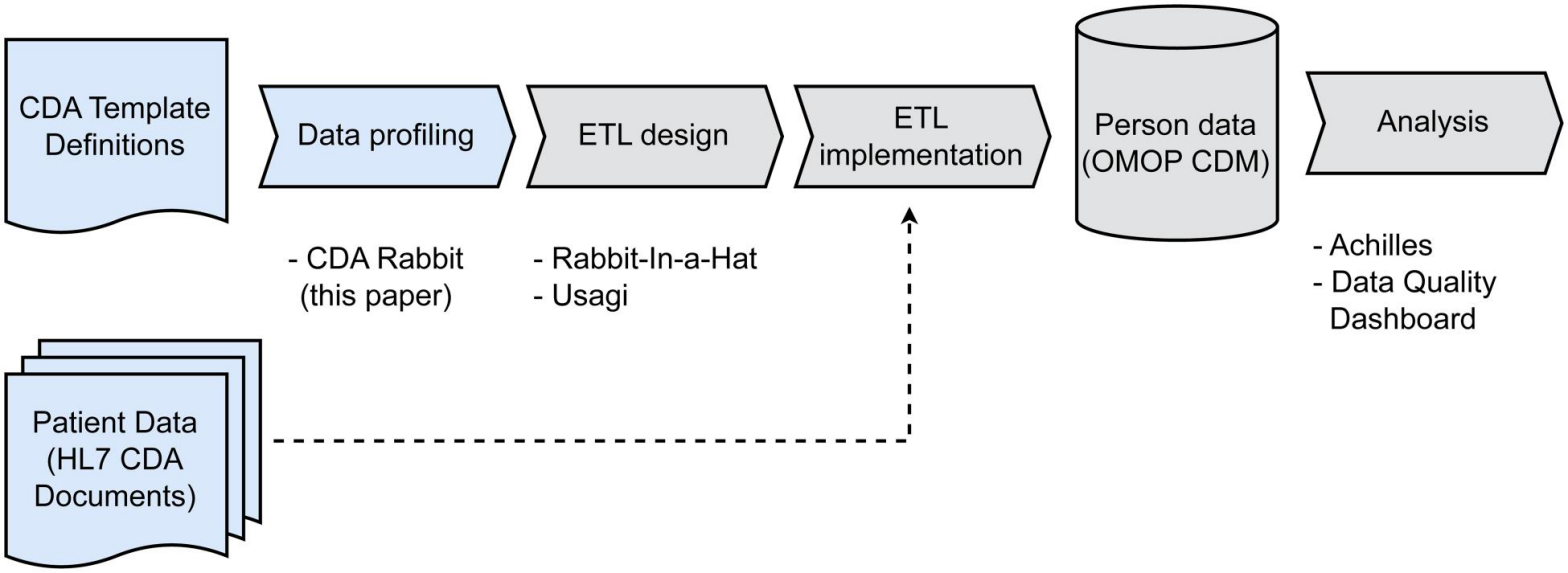
In contrast to WhiteRabbit (compare Figure 1), CDA Rabbit uses CDA Template definitions as input for data profiling. Subsequent steps remain unchanged. Note that actual CDA documents containing patient data are required at a later stage, namely ETL implementation, testing, and execution. Abbreviation: CDA, Clinical Document Architecture.

As OHDSI’s WhiteRabbit tool only supports relational data sources as input, we created an alternative tool “CDA Rabbit,” which implements the aforementioned methodology. Clinical Document Architecture Rabbit parses a CDA Template repository file and our set of default CDA R-MIM-based mappings to generate a project file that serves as input for OHDSI’s Rabbit-In-a-Hat tool. This project file then contains all template definitions and treats them as if they were tables in a relational data model, displaying elements contained in a template as attributes of these “tables.” Derived default mappings are also included, along with the abovementioned extracted information. This allows Rabbit-In-a-Hat to be applied for ETL design as usual, and to follow the common approach to ETL design imposed by the OHDSI community. This adapted OMOP ETL process is shown in Figure 3.

**Figure 4.**
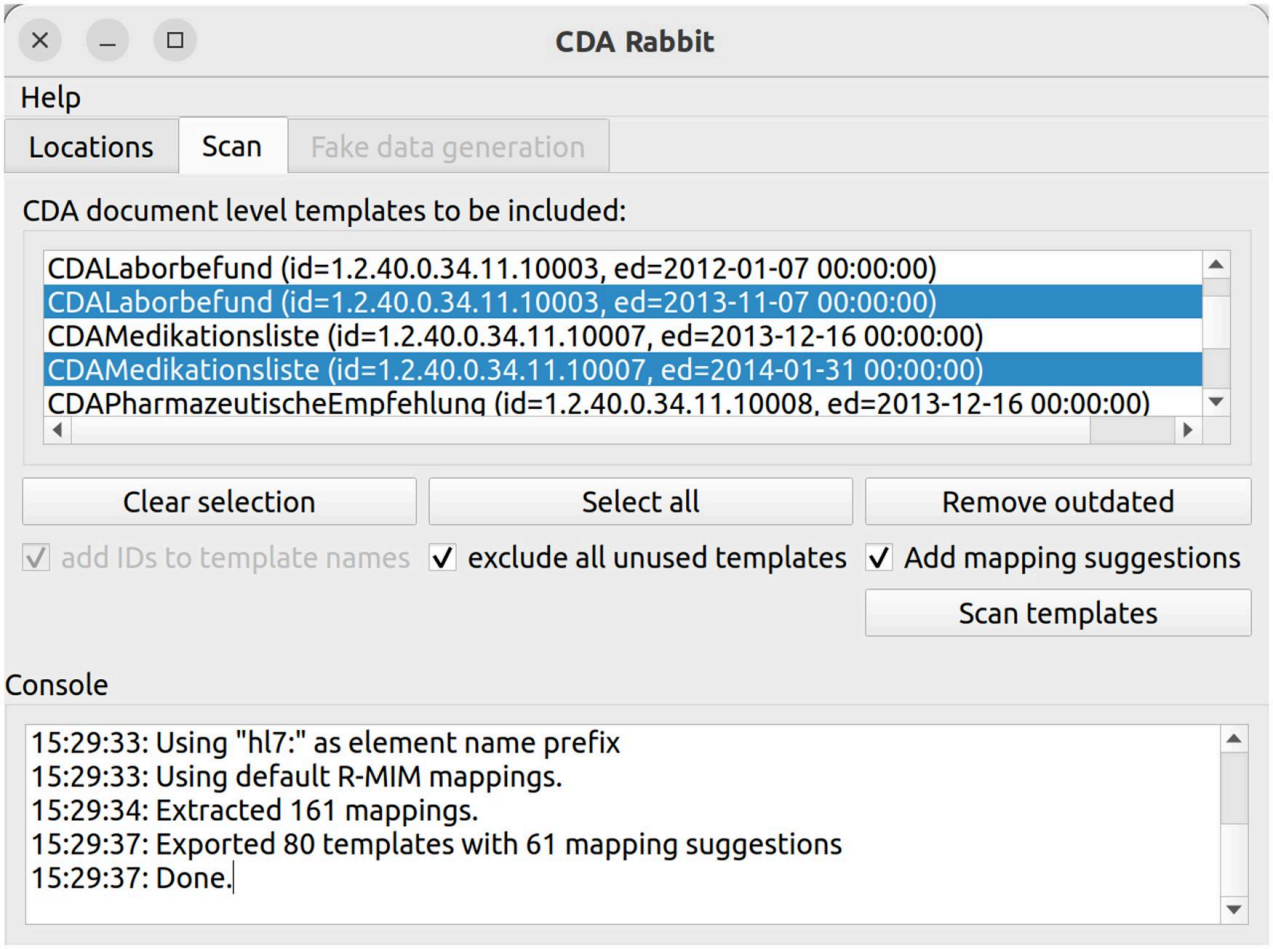
Clinical Document Architecture (CDA) Rabbit shows all document level CDA Templates contained within the loaded CDA Template repository file. After selecting CDA Templates and executing the scan, all relevant documents, section, and entry level CDA Templates, their properties, and default mappings are exported to a Rabbit-In-a-Hat project file. Abbreviation: CDA, Clinical Document Architecture.

**Figure 5.**
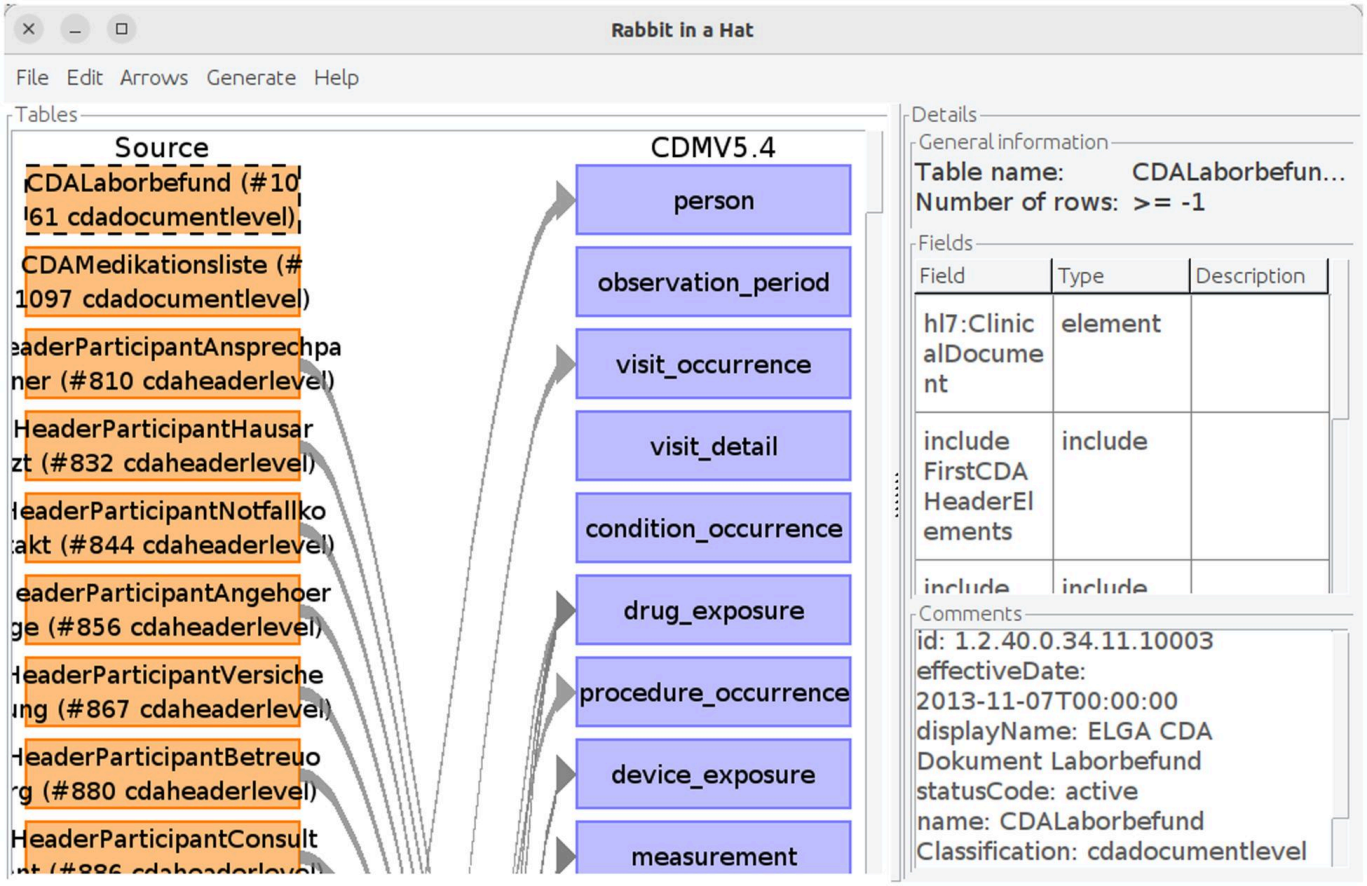
Rabbit-In-a-Hat with a loaded project file, generated by CDA Rabbit. The depicted arrows between CDA Templates (orange) and the OMOP CDM Tables (blue) are suggested default mappings derived from the semantics of the CDA R-MIM that may be further refined by the user. Additional information (eg, template identifiers, paths, elements) extracted from the selected CDA Template (dashed box) is displayed on the right. Abbreviations: CDA, Clinical Document Architecture; OMOP, Observational Medical Outcomes Partnership; R-MIM, Refined Message Information Model.

Clinical Document Architecture Rabbit was developed as a Python v3.10 application with a Qt5 user interface. It recreates the WhiteRabbit interface, allows for CDA Template repository files to be loaded and additionally allows to select document level CDA Templates (see Figure 4). The sources, along with precompiled executables for Windows and Linux, are available as open source software from our Git repository: mimapps.meduniwien.ac.at/gitlab/omop/cdarabbit. The output of CDA Rabbit is then loaded into OHDSI’s Rabbit-In-a-Hat tool (see Figure 5), where it forms the basis for further ETL design.

## Results

The default mappings, which we derived from the semantics of the CDA R-MIM classes, are available in spreadsheet format from (see Supplementary Material).

We tested our approach by means of a set of 430 anonymized ELGA CDA documents of type *Physician Discharge Summary* (330) and *Laboratory Report* (100). We loaded the 2 corresponding document level CDA Templates and all contained subtemplates (in total 13 CDA Templates) into our tool CDA Rabbit. Originating from the default mappings suggested by CDA Rabbit, we mapped the CDA Templates to 10 CDM tables (namely *observation_period, visit_occurrence, condition_occurrence, drug_exposure, measurement, observation, specimen, location, care_site,* and *provider*). We distinguish between (1) table level mappings, which are mappings between CDA Templates and CDM Tables and (2) field level mappings, which map elements within a template to specific fields in a CDM Table. Of all table level mappings used in our ETL definition document, 60% were correctly suggested by the default mapping process; 41% of all field level mappings were correctly suggested. No default mapping was found to be incorrect. The remaining mappings were then manually added using the Rabbit-In-a-Hat tool. Our tool also identified 12 source vocabularies used in the CDA Templates that needed to be mapped to OMOP’s standard vocabularies.

As an example, CDA entry level Template *VitalparameterEntry*, which is used to capture vital signs, was mapped to the CDM’s *measurement* table according to the suggested CDA R-MIM derived default mapping. Besides the *Physician Discharge Summary*, this template is also contained in the ELGA *Nursing Discharge Summary* and the *Nursing Transfer Note* document types. The mapping can thus be reused for the transformation of further CDA document types. The template’s element “code” is bound to a value set of codes from the LOINC terminology, which is also the standard vocabulary for measurements in OMOP, so no further vocabulary mapping is needed.

Using a Python-based implementation of the transformation,^28^ the data contained in the 430 CDA documents populated 9 CDM tables (as mentioned above, table *drug_exposure* was not populated since our 430 CDA documents did not contain data for the optional medication template within document type *Physician Discharge Summary*) with a total of 4041 entries. The data quality assessment using OHDSI’s DataQualityDashboard accredited 99% of the tests as passed. The remaining failed tests could be attributed to implausible source data.

## Discussion and future work

Previous work has shown the feasibility of transforming CDA documents into OMOP CDM,^15^^,^^16^^,^^19^ commonly using an intermediate relational database,^12^^,^^17^^,^^18^ which requires a complex database design process. Other researchers employ absolute XPath expressions as selectors within CDA documents,^15^^,^^19^ which hinders the reuse of existing transformations for CDA components. In particular, CDA entry level components are frequently designed in a highly reusable way. As an example, a CDA component for medication supplies is contained in ELGA medication dispensing, medication list, and hospital discharge summary documents. Its transformation to OMOP’s CDM table *drug_exposure* is thus amenable for reuse.

Applying CDA Templates as mapping sources enables the reuse of transformations, but, to our knowledge, has not been discussed in literature, apart from our previous paper.^28^ Clinical Document Architecture Rabbit discovers all possible locations where a given template can “appear” within a CDA instance and also generates the relative XPath expressions for elements within a template. These locators can be utilized in the actual ETL implementation.

Voss et al studied prerequisites for successful ETL projects across 25 sites and found that data access difficulties frequently delay ETL projects.^22^ Our proposed method decouples ETL design from access to CDA instance data. As it just requires CDA Template definitions, ETL design work could start before gaining access to the actual CDA instances.

Ong et al provides a multidimensional framework of common ETL challenges.^29^ Applying CDA Templates as the basis of ETL contributes to a better “knowledge of source data” and a comprehensive and computable “documentation of the source data model,” while avoiding “source data accessibility” issues during ETL design.

The proposed approach could be further extended to allow automatic code generation, exploiting the standardized nature of CDA Templates and their CDA R-MIM foundation. Explicit references to known vocabularies, the expressiveness of cardinalities, and default values could allow boilerplate code to be initialized. By using vocabulary bindings together with existing vocabulary mappings and OMOP’s assignment of standardized codes to specific domains, vocabulary constraints can be used to map CDA elements to specific CDM tables. For example, a vocabulary binding to the Anatomical Therapeutic Chemical (ATC) Classification System for a CDA element suggests that a mapping to OMOP’s *drug_exposure* table is appropriate, since all ATC codes are related to OMOP’s drug domain.

The fact that CDA Rabbit purely refers to CDA Template definitions, but ignores the actual CDA instances, represents a current limitation. Analysis and collection of sample data from CDA instances during the source data characterization process could further streamline the ETL design process. Similar to WhiteRabbit’s functionality, this could provide summarized frequencies, data modalities, and statistics on missing data. It could also provide insights into vocabularies and code usage.

Another limitation of CDA Rabbit is that it cannot resolve cyclic template dependencies. The HL7 Template Standard itself does not restrict the usage of cyclic dependencies among CDA Templates. This nesting can occur and is difficult to incorporate into the ETL design. Our current implementation detects cyclic dependencies but does not resolve them. However, we did not detect a single instance of a cyclic template dependency in the ELGA template definitions, that is, they seem to occur rarely.

The HL7 Template Standard can be used to restrict any XML-based data representation, including XML formatted HL7 Fast Healthcare Interoperability Resources (FHIR). Nevertheless, a FHIR-specific mechanism named profiling is typically employed to constrain FHIR resources. As the transformation of FHIR source data to OMOP CDM is another obvious goal and has been demonstrated to be feasible,^30^ an extension of our work could focus on adapting CDA Rabbit to FHIR profiles as source structure definitions.

The introduction of common data models alone is not sufficient to mitigate the effects of source data heterogeneity on subsequent analysis.^31^ Future work should also investigate the heterogeneity of different CDA documents that are constrained by the same CDA Template. The transformation of any clinical data into common data models implies the potential for information loss. Observational Medical Outcomes Partnership projects often require the alignment of local terminologies with standardized ones. This vocabulary mapping can result in the loss of granularity, omission of information and potentially impact downstream analyses. However, standardized CDA documents are often already based on vocabulary bindings to standardized vocabularies, making them a very suitable data source for OMOP projects. The ELGA CDA documents mentioned above contain vocabulary bindings to several standardized vocabularies used in OMOP, making vocabulary mappings entirely obsolete (eg, laboratory measurements as LOINC codes).

## Conclusion

In this work, we have presented a novel approach to transform HL7 CDA documents into OMOP CDM by leveraging CDA Templates and the CDA R-MIM meta-model. In contrast to earlier work that used absolute XPath expressions, it allows easy reuse of transformations of CDA components. In addition, our approach enables the ETL design to start without requiring access to the actual CDA instance data at this stage. It thus mitigates data access delays that are a common obstacle in ETL projects.

For an easy application of our approach, we have implemented a tool named CDA Rabbit. It integrates seamlessly into the OHDSI toolchain by generating Rabbit-In-a-Hat project files directly from CDA Template definitions. Considering the semantics of the CDA R-MIM classes and the OMOP CDM tables, it generates likely default mappings in the project file, which are then suggested to the user of Rabbit-In-a-Hat. We tested our approach by importing 13 CDA Templates, which define the structure and vocabulary of our test set of 430 anonymized ELGA CDA documents, into CDA Rabbit. Originating from the suggested default mappings, we defined the transformations to 10 tables of the OMOP CDM. Data quality assessment of the resulting OMOP data showed a 99% pass rate, indicating a robust and accurate data transformation. Clinical Document Architecture Rabbit and related resources are publicly available at: https://mimapps.meduniwien.ac.at/gitlab/omop/cdarabbit

Areas for further research are automating code generation based on CDA Templates, handling cyclic dependencies within template repositories, adding data profiling capabilities on the actual CDA instances, and considering FHIR profiles as source structure definitions.

Overall, this work contributes to improving semantic interoperability and secondary use of health data. By providing a robust framework for transforming routine CDA data into a research-friendly format, we support the growing need for large-scale clinical research and cross-organizational data sharing, ultimately contributing to better health-care outcomes.

## Supplementary Material

ooaf022_Supplementary_Data

## Data Availability

A human-readable representation of HL7 Template Definitions, among other information, for Austria’s national EHR system ELGA can be accessed through the publicly available ART-DECOR tool hosted at: https://elga.art-decor.org/. The XML representation, including HL7 Template Definitions under the “<rules>” element can be obtained from: https://art-decor.org/decor/services/RetrieveProject. The set of anonymized ELGA CDA documents used to test our ETL implementation cannot be shared publicly (see Acknowledgments), but a (smaller) set of officially published ELGA CDA instances can be obtained from: https://gitlab.com/elga-gmbh/cda-beispielbefunde. Source code and compiled versions of the proposed CDA Rabbit tool can be obtained from: https://mimapps.meduniwien.ac.at/gitlab/omop/cdarabbit.
